# Cardiomyocytes cultured on mechanically compliant substrates, but not on conventional culture devices, exhibit prominent mitochondrial dysfunction due to reactive oxygen species and insulin resistance under high glucose

**DOI:** 10.1371/journal.pone.0201891

**Published:** 2018-08-23

**Authors:** Masaki Morishima, Kazuki Horikawa, Makoto Funaki

**Affiliations:** 1 Clinical Research Center for Diabetes, Tokushima University Hospital, Tokushima, Japan; 2 Division of Bioimaging, Institute of Biomedical Sciences, Tokushima University Graduate School, Tokushima, Japan; Stellenbosch University, SOUTH AFRICA

## Abstract

**Rationale:**

Diabetes causes cardiac dysfunction, and understanding of its mechanism is still incomplete. One reason could be limitations in modeling disease conditions by current *in vitro* cardiomyocyte culture. Emerging evidence suggests that the mechanical properties of the microenvironment affect cardiomyocyte function. Nevertheless, the impact of high glucose on cardiomyocytes cultured on substrates whose stiffness matches that of the heart (approximately 15 kPa) is untested.

**Objective:**

To test the hypothesis that cardiomyocytes cultured in microenvironments that mimic the mechanical properties of those for cardiomyocytes *in vivo* may reproduce the pathophysiology characteristics of diabetic cardiomyocytes *ex vivo*, such as the morphological appearance, ROS accumulation, mitochondrial dysfunction, apoptosis and insulin-stimulated glucose uptake.

**Methods and results:**

Isolated neonatal rat cardiomyocytes were seeded on 15 kPa polyacrylamide (PAA) gels, whose stiffness mimics that of heart tissues, or on glass coverslips, which represent conventional culture devices but are unphysiologically stiff. Cells were then cultured at 5 mM glucose, corresponding to the normal blood glucose level, or at high glucose levels (10 to 25 mM). Cytoskeletal disorganization, ROS accumulation, attenuated mitochondrial membrane potential and attenuated ATP level caused by high glucose and their reversal by a ROS scavenger were prominent in cells on gels, but not in cells on coverslips. The lack of response to ROS scavenging could be attributable to enhanced apoptosis in cells on glass, shown by enhanced DNA fragmentation and higher caspase 3/7 activity in cells on glass coverslips. High-glucose treatment also downregulated GLUT4 expression and attenuated insulin-stimulated glucose uptake only in cells on 15 kPa gels.

**Conclusion:**

Our data suggest that a mechanically compliant microenvironment increases the susceptibility of primary cardiomyocytes to elevated glucose levels, which enables these cells to serve as an innovative model for diabetic heart research.

## Introduction

Contractile dysfunction of the heart due to chronic hyperglycemia, independent of coronary artery disease, is a major challenge in tackling diabetes [1, 2]. Intensive research has been conducted to elucidate its mechanism, and one probable cause is excess accumulation of reactive oxygen species (ROS) [3–5]. In fact, ROS levels are increased in cultured cardiomyocytes exposed to high glucose concentrations and in diabetic animal models [3]. Accumulated ROS alter cellular signaling and damage cells, which includes progressive deterioration of mitochondrial function that leads to oxidative stress [6–8]. The cytoskeletal system, including the actomyosin system, plays a pivotal role in cardiomyocyte contractility [9]. However, *in vitro* study to reveal whether or not high-glucose-induced ROS affect the cytoskeletal system has been insufficient, mainly due to limitations in modeling cardiomyocyte function *in vitro*.

One of the pathological features of cardiac muscle in type 2 diabetes is attenuated insulin-stimulated glucose uptake [10]. Glucose transporter 4 (GLUT4) is expressed in skeletal/cardiac muscle and adipocytes and plays a major role in insulin-stimulated glucose uptake; insulin stimulation triggers a series of signal transduction to translocate intracellular GLUT4-containing vesicles to the plasma membrane, where GLUT4 is engaged in glucose uptake into cells. In the diabetic heart, GLUT4 expression is downregulated, which contributes to impaired insulin-stimulated glucose uptake [11].

*In vitro* cell-based assays would provide easier access and operability, but it appears that they cannot match the complexity of *in vivo* events [12]. Thus, *in vitro* research very frequently has been met with inconsistent results from those of *in vivo* assays/human trials. One huge limitation of *in vitro* assays is that cells cultured *ex vivo* mimic only a part of the cellular functions of their relevant cells *in vivo*. Numerous efforts have been made to develop culture conditions reproducing *in vivo* cellular functions [13]. Emerging evidence suggests that not only the biochemical properties of the microenvironment but also its biophysical properties affect cellular functions. One biophysical property that has recently drawn much attention is the stiffness of the microenvironment, and hydrogels are widely used to mimic the stiffness of the microenvironment *in vivo* [12]. The heart is one organ whose functions are closely associated with such mechanical property [14–17].

In this study, we hypothesized that culturing primary cardiomyocytes on substrates whose stiffness mimics that of the heart may enable reproduction of the pathophysiology of diabetic cardiomyocytes *ex vivo*. To probe this hypothesis, neonatal rat cardiomyocytes were seeded on 15 kPa polyacrylamide gels. Here, we provide evidence that high-glucose treatment reversibly causes ROS-dependent mitochondrial dysfunction and insulin resistance in cardiomyocytes. Thus, primary cardiomyocytes cultured on mechanically compliant substrates could serve as one of the best *in vitro* cell-based assays for studying the diabetic heart *ex vivo*, since the functions of these cells may be closest to those of cardiomyocytes *in vivo*.

## Materials and methods

### Reagents

Reagents were obtained from Sigma Aldrich (St. Louis, MO) or WAKO (Osaka, Japan) unless otherwise indicated. Triton X-100 was purchased from MP Biomedicals (Aurora, OH). Fetal bovine serum was obtained from Biosera (Biosera, Nuaillé, Chile). Collagenase type IV was purchased from Worthington (Lakewood, NJ). H_2_DCF-DA (2',7'-dichlorodihydrofluorescein diacetate), JC-1 (5,5',6,6'-tetrachloro-1,1',3,3'-tetraethylbenzimidazolocarbocyanine iodide), ProLong Diamond Antifade Mountant with 4',6-diamidino-2-phenylindole dihydrochloride (DAPI), Alexa Fluor 488-conjugated phalloidin and MitoTracker Red (CMXRos) were from Molecular Probes (Eugene, OR). Alexa Fluor-conjugated secondary antibodies were from Cell Signaling (Danvers, MA). Anti-α-actinin antibodies were from Sigma (A7811). All reagents from commercial sources were of analytical grade.

### Preparation and culture of neonatal rat cardiomyocytes

The experimental protocol was approved by the Ethics Review Committee for Animal Experimentation at Tokushima University. Neonatal rat cardiomyocytes were prepared from 1- to 3-day old Wistar rat ventricles by digestion with type 4 collagenase, as previously described [18]. Briefly, enzymatically isolated cells suspended in Dulbecco’s Modified Eagle’s Medium (DMEM, 1000 mg/L glucose) were plated on tissue culture dishes and incubated for 40 min to allow attachment of fibroblasts and endothelial cells to the bottom of dishes. Floating cardiomyocytes were then collected, plated onto either polyacrylamide (PAA) gels or glass coverslips at a concentration of 1.5×10^5^ cells/well, and cultured in DMEM supplemented with 5% fetal bovine serum, 10 mM HEPES, and kanamycin (100 IU). After 24 h of culture, >70% of the cells adhered to the substrates and started to exhibit spontaneous beating (data not shown).

### PAA (polyacrylamide) gel preparation

First, 12% acrylamide and 0.15% bisacrylamide (Bio-Rad Laboratories, Hercules, CA) in phosphate buffered saline (PBS) (pH 7.4) were used to prepare 15 kPa gels, and they were polymerized on glass coverslips, as described previously [19]. The surface of PAA gels was coated with a mixture of 0.05 mg/ml fibronectin and 0.1 mg/ml collagen type I by binding them to N-sulfosuccinimidyl-6-(4’-azido-2’-nitrophenylamino) hexanoate (0.5 mg/ml in 50 mM HEPES buffer pH 8) (Thermo Fisher Scientific), which was covalently linked to the PAA gels, as described previously [20]. Glass coverslips were submerged in a mixture of fibronectin and collagen type 1 solution to coat their surface with extracellular matrix (ECM) ligands. The gels or glass coverslips were placed into a six-well plate, which was pre-coated with 1% agarose gel to prevent adhesion of cells outside the gels or coverslips.

### Cell culture and exposure of cardiomyocytes to high-glucose concentrations

Cardiomyocytes were incubated for 24 h to establish adhesion to either PAA gel substrates or glass coverslips, and were subsequently treated with various concentrations of glucose (5, 10, 15 or 25 mM) in the presence or absence of ROS scavengers, such as N-acetyl cysteine (NAC; 0.25 and 1 mM, Sigma) and manganese (III) tetrakis (4-benzoic acid) porphyrin chloride (MnTBAP; 0.1 mM, Sigma) for 24 h. To investigate the effect of removing accumulated ROS, cardiomyocytes were first exposed to a high level of glucose for 24 h, followed by treatment with 5 mM glucose in the presence or absence of NAC. Where indicated, cells were treated with mannitol (WAKO), instead of glucose, to evaluate the effect of high osmolality, which could also be induced by high-glucose treatment.

### Imaging

Live imaging of cells stained with H_2_DCFDA and JC-1 was performed using a confocal microscope system (A1R, Nikon, Tokyo, Japan) equipped with a PlanFluor 40X objective lens (N.A. 0.6, W.D. 3.7–2.7 mm, Nikon) and excitation lasers (488 and 561 nm, Melles Griot). To measure the ROS level, a stage incubator (37°C and 5% CO_2_) was utilized. For immunocytochemical study and TUNEL assays, images were obtained with an AxioImager M2 epifluorescence microscope equipped with 20X and 40X objective lens, an AxioCam HR camera, an excitation filter BP379-401; dichromatic filter FT 420; emission 435–485; under the control of AxioVision rel. 4.8 software (Carl Zeiss, Jena, Germany). Images were saved in TIFF format and analyzed by ImageJ software (Wayne Rasband, National Institutes of Health). For quantification, images were taken of at least five randomly selected fields for each sample, and fluorescence intensity in at least 24 cells was quantified per sample in three independent experiments. Areas without cells in each sample were considered as background and their intensity was subtracted from fluorescence intensity.

### Detection of intracellular ROS accumulation

Intracellular ROS accumulation was detected using H_2_DCFDA, whose green fluorescence signal is increased by its oxidation by ROS. Cells were incubated with 2 μM H_2_DCFDA for 50 minutes at 37°C and rinsed with HEPES buffer twice before observation.

### Assessment of mitochondrial membrane potential

JC-1 dye, whose fluorescence emission shifts from red to green when depolarized, was used to quantify mitochondrial membrane potential. After high-glucose treatment with or without NAC (0.25, 1 mM), cells were incubated with JC-1 for 30 min at 37°C, and fluorescence intensity was measured on a confocal laser microscope. Data were quantified by calculating the red fluorescence intensity/green fluorescence intensity ratio. In fixed cells, cells were loaded with 0.2 μM of fluorochrome MitoTracker Red (CMXRos) at 37°C for 30 min, fixed by 4% paraformaldehyde (Wako) for 10 min and imaged on an AxioImager M2 epifluorescence microscope (Carl Zeiss). Data were quantified by calculating the reduction in the area of CMXRos staining, which is indicative of a cell whose mitochondria have reduced membrane potential [21].

### Immunocytochemistry procedure

After 48 h in culture, cells were fixed with 4% paraformaldehyde (Wako) for 10 min and then washed three times with PBS. Cells were permeabilized with 0.1% Triton X-100 in PBS for 10 min and washed three times for 5 min in PBS. Cells were blocked with 10% BSA for 1 h and then incubated for 1 h at room temperature with mouse monoclonal anti-α-actinin (1:400) antibodies followed by corresponding fluorescently-labeled secondary antibodies to visualize sarcomere assembly or fluorescently-labeled Alexa Fluor^TM^ 488 phalloidin (Invitrogen) to visualize F-actin. After several washes, the samples were air-dried, mounted with a drop of ProLong Diamond Antifade Mountant with DAPI (Molecular Probes) and subjected to microscopy.

### TUNEL assay

To count the number of apoptotic cells, TUNEL assays were performed using an *In Situ* Cell Death Detection Kit (Roche, Indianapolis, IN, USA) according to the manufacturer’s instructions. Cells were co-stained with DAPI to visualize the nucleus. Nuclei that were double-labeled with DAPI and TUNEL were considered positive, and the percentage of positive nuclei among the total number of nuclei, which was represented by the number of DAPI-stained nuclei, was calculated.

### Measurement of intracellular ATP level

Cardiomyocytes were cultured on either 15 kPa PAA gels or glass coverslips at a density of 1.5 × 10^5^ cells per sample in DMEM medium supplemented with 5% FBS, 10 mM HEPES, and kanamycin (100 IU) for 24 h to allow cell adhesion. Cells were subsequently treated with 5 or 15 mM glucose in the presence or absence of NAC (1 mM) for 24 h. Intracellular ATP levels were measured using a CellTiter-Glo Luminescent Cell Viability Assay kit (Promega, Madison, WI, USA), according to the manufacturer’s instructions. Luminescence was measured using a Glomax Multi Plus Detection System (Promega, Madison, WI, USA). The amount of ATP was calibrated by the number of cells.

### Caspase3/7 activity assay

Caspase activity was detected using a Caspase-Glo® 3/7 assay kit (Promega, Madison, WI, USA). Briefly, cardiomyocytes were cultured at 1.5 × 10^5^ cells per sample on either 15 kPa PAA gels or glass coverslips in DMEM medium supplemented with 5% FBS, 10 mM HEPES, and kanamycin (100 IU). After 24 h of culture to allow cell adhesion, cells were treated with either 5 or 15 mM glucose in the presence or absence of NAC (1 mM) for 24 h. As a positive control, cells were treated with staurosporine (1 μM) for 24 h to activate caspase-3. Caspase-3/7 activity was determined according to the manufacturer’s instructions. Luminescence was measured using a Glomax Multi Plus Detection System (Promega, Madison, WI, USA), and caspase 3/7 activity was calibrated by the number of cells.

### Glucose uptake assay

Glucose uptake was measured as described previously [22]. Briefly, after exposing cells on either glass coverslips or 15 kPa PAA gels to various concentrations of glucose in the absence or presence of 1 mM NAC for 24 h, they were serum-starved for 3 h in DMEM containing 0.2% bovine serum albumin, followed by glucose starvation in Krebs-Ringer phosphate buffer (in mM: 137 NaCl, 4.7 KCl, 10 sodium phosphate (pH 7.4), 0.5 MgCl_2_, 1 CaCl_2_) for an additional 45 min. Cells were either left untreated or stimulated with 100 μM insulin for 15 min at 37°C. Uptake of 2-deoxyglucose was measured in cardiomyocytes using a glucose uptake-Glo^TM^ assay kit (Promega, Madison, WI, USA) and luminescence intensity (Relative light unit, RLU) was measured according to the manufacturer’s instructions.

### Real time PCR

Single-stranded cDNA was synthesized from 500 ng of total RNA using a Transcriptor First Strand cDNA Synthesis Kit (Roche Molecular System Inc., Alameda, CA, USA). Real-time PCR was performed on a StepOnePlus real time PCR system (Applied Biosystems, Foster City, CA) using Fast SYBR Green Master Mix (Applied Biosystems). Primers used were; (GLUT1; NM_138827) forward 5’-acgtccattctccgtttcac-3’ and reverse 5’-tcccacggccaacataag-3’; (GLUT4; NM_012751) forward 5’-ttgcagtgcctgagtcttctt-3’ and reverse 5’-ccagtcactcgctgctga-3’; (β-actin; NM_031144.3) forward 5’-gagcgcggctacagctt-3’ and reverse 5’-tccttaatgtcacgcacgattt-3’. Data were calculated by 2^–ΔΔ^CT and presented as fold change in transcripts for each glucose transporter gene and normalized to β-actin (defined as 1.0-fold).

### Statistics

Experiments were performed at least three times. Statistical analysis was conducted using SigmaPlot 12.5 (SigmaPlot version 12.5 –Systat Software, Inc., London, UK). Values are mean ± S.D. Values of p were calculated using unpaired Student’s t-test for differences between two groups, and one-way ANOVA for differences among multiple groups. Values of *p* < 0.05 were considered statistically significant.

## Results

### High-glucose treatment disorganized cytoskeletal structures in cardiomyocytes on mechanically compliant 15 kPa PAA gels

**Fig 1:**
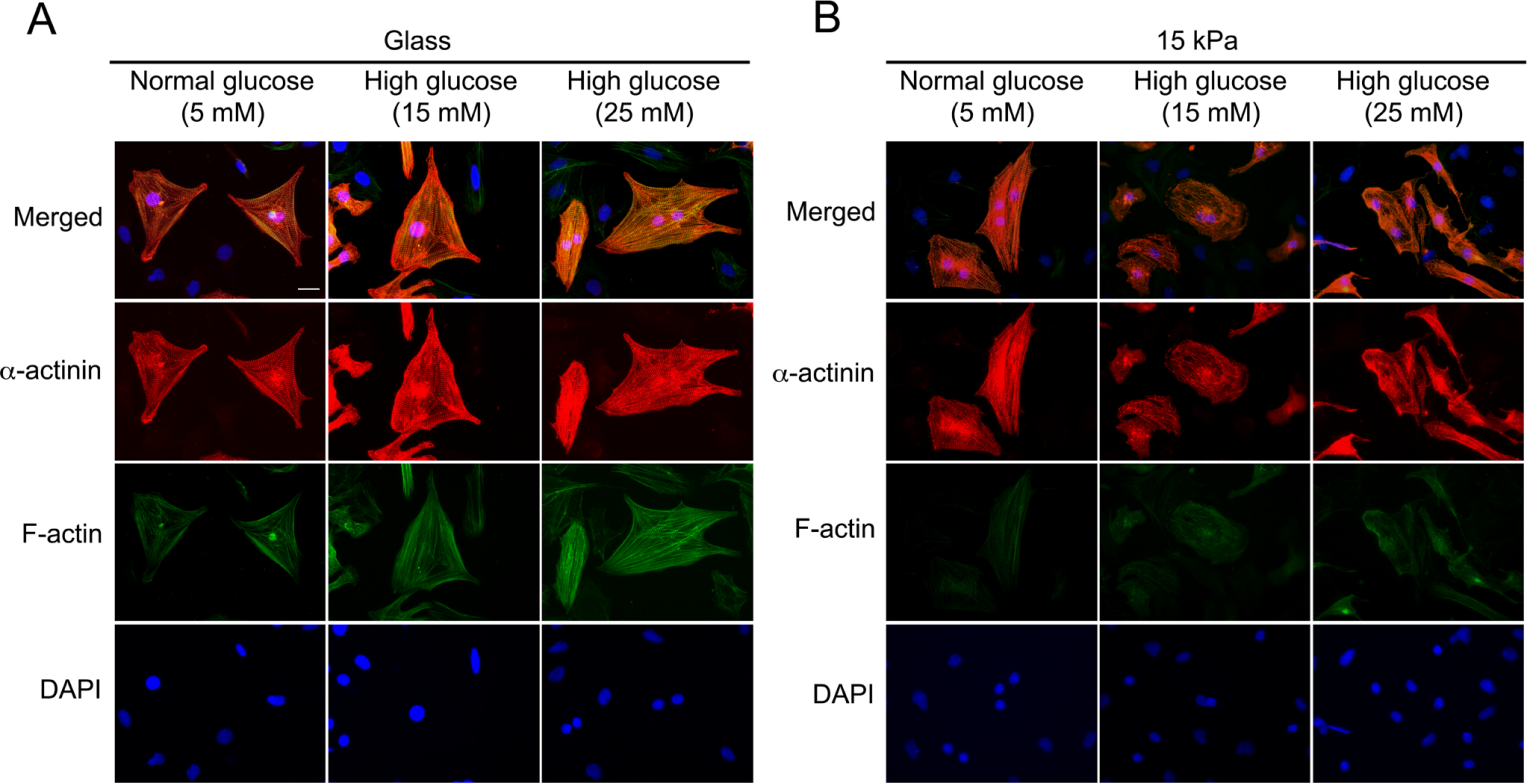
Effect of high-glucose treatment on sarcomere structures/cytoskeletal system. Cardiomyocytes were seeded on either glass coverslips (**A**) or 15 kPa PAA gels (**B**). Cells were exposed to 5, 15 or 25 mM glucose for 24 h. Sarcomere structures were identified by staining for α-actinin (red), F-actin structures were stained with phalloidin (green), and nuclei were stained with DAPI (blue). Representative images are shown. Similar results were obtained from three other independent experiments. Scale bar = 20 μm.

Cardiomyocytes were cultured either on 15 kPa PAA gels, whose stiffness matched that of the heart, or on glass coverslips representing a conventional stiff culture device. F-actin structures and sarcomeres were visualized by staining with phalloidin and anti-α-actinin antibodies, respectively. When cells were cultured with medium containing 5 mM glucose, which corresponds to a normal glucose level, cells on 15 kPa PAA gels elongated and exhibited sarcomeres aligned parallel to the long axis of the cells, as reported previously [14]. On the other hand, cardiomyocytes seeded on glass coverslips, which are an order of magnitude stiffer than the heart, spread out broadly and exhibited randomly aligned sarcomeres **(Fig 1A and 1B)**.

To investigate whether a diabetic condition would influence these cytoskeletal structures, cardiomyocytes were subjected to high-glucose treatment. As shown in **Fig 1A**, cardiomyocytes seeded on mechanically non-compliant glass coverslips failed to show any change in F-actin or sarcomere structures by high-glucose treatment (15 or 25 mM glucose). Surprisingly, cardiomyocytes seeded on 15 kPa PAA gels exhibited disorganized F-actin structures and sarcomeres under high-glucose treatment **(Fig 1B)**. Treatment with 15 mM mannitol failed to disorganize cytoskeletal structures in cardiomyocytes on 15 kPa PAA gels **(S1A Fig)**. Thus, the disorganization of cytoskeletal structures caused by high glucose was independent of alterations in osmolality. These results suggest that high glucose deregulates cytoskeletal structures in cardiomyocytes when these cells are on mechanically compliant substrates.

### Enhanced ROS accumulation by high-glucose treatment and its prevention by ROS scavenger in cardiomyocytes on 15 kPa gels

**Fig 2:**
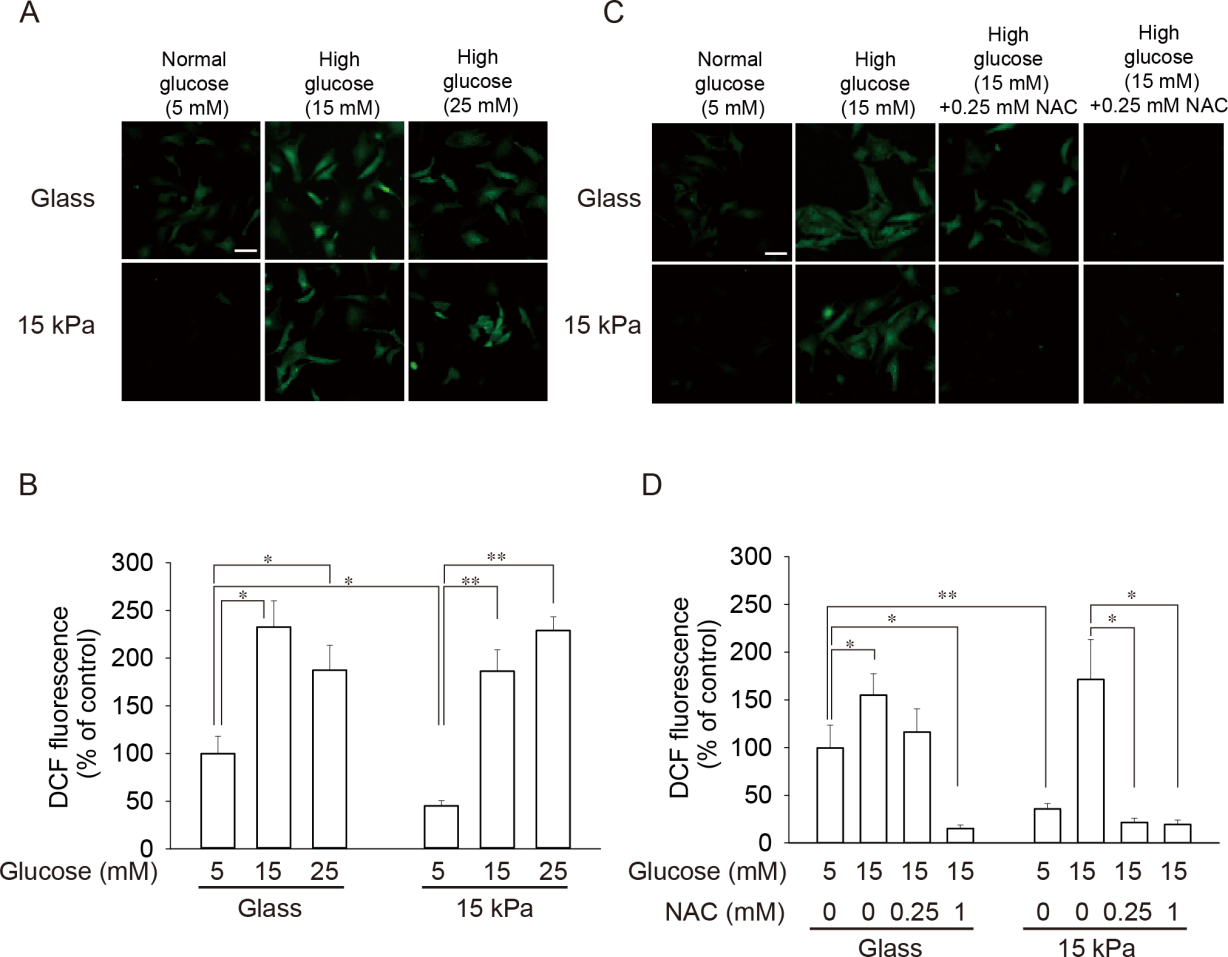
Low basal level of ROS and enhanced prevention of high-glucose-induced ROS accumulation by ROS scavenger in cells on 15 kPa gels compared to cells on glass. Cardiomyocytes were seeded on either glass coverslips (Glass) or 15 kPa PAA gels (15 kPa). (**A, B**) Cells were incubated with 5, 15 or 25 mM glucose for 24 h and stained with H_2_DCFDA. Representative images (**A**) and quantitative results are shown (**B**). (**C, D**) Cardiomyocytes were incubated with 5 or 15 mM glucose in the absence or presence of 0.25 or 1 mM NAC for 24 h. Cells were stained with H_2_DCFDA. Representative images (**C**) and quantitative results are shown (**D**). (**B, D**) Fluorescence intensity of cells on glass coverslips treated with 5 mM glucose was set as 100%, and data are expressed as mean ± SD (n = 24). Scale bar = 50 μm. *p < 0.05, **p < 0.01.

To investigate the effect of high-glucose treatment on the level of oxidative stress, we measured the amount of reactive oxygen species (ROS) accumulated in cardiomyocytes. As shown in **Fig 2A and 2B**, ROS level in cells exposed to 5 mM glucose was significantly lower in cells on 15 kPa gels than in those on glass. High-glucose treatment caused ROS accumulation in cells on 15 kPa PAA gels at a level that was comparable to that in cells on glass treated with high glucose (**Fig 2A and 2B**). The effect of scavenging ROS on high-glucose-induced ROS accumulation was investigated by exposing the cells to high glucose in the absence or presence of a ROS scavenger, N-acetyl cysteine (NAC). As shown in **Fig 2C and 2D**, NAC abolished ROS accumulation induced by 15 mM glucose both in cells on 15 kPa gels and on glass. However, prevention of ROS accumulation was apparent at a lower concentration of NAC in cells on 15 kPa gels compared to those on glass. Unlike glucose, mannitol failed to cause accumulation of ROS in cells on 15 kPa gels, demonstrating that ROS accumulation in these cells was not due to osmotic change **(S2 Fig)**. These results indicate that ROS accumulation is higher in cells on glass coverslips, even at a glucose level corresponding to a normal blood glucose level. Furthermore, cells on 15 kPa PAA gels are more sensitive to increased glucose levels with respect to ROS accumulation, and a scavenger like NAC can prevent ROS accumulation more effectively in these cells. Addition of NAC also restored the disorganized cytoskeleton caused by high glucose in cells on 15 kPa PAA gels, suggesting involvement of ROS in high-glucose-induced alteration of the cytoskeleton **(S3 Fig)**.

### Attenuated mitochondrial membrane potential and intracellular ATP level under high glucose and their prevention by ROS scavenger were observed only in cardiomyocytes on 15 kPa gels

**Fig 3:**
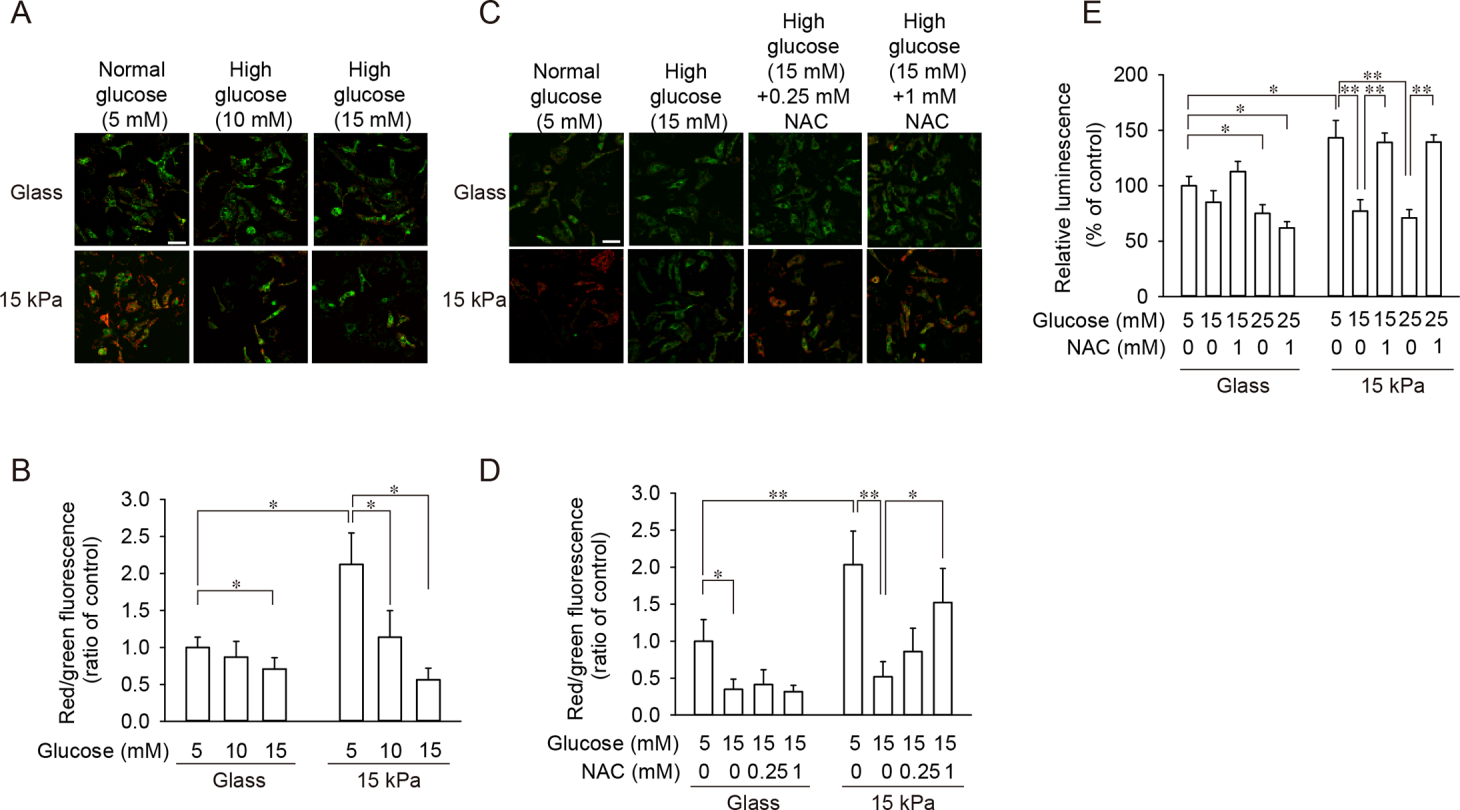
Moderately high level of glucose (10–15 mM) attenuated mitochondrial membrane potential and intracellular ATP level, which was prevented by ROS scavenger in cells on 15 kPa gels, but not in cells on glass coverslips. **(A-D)** Cardiomyocytes were seeded on either glass coverslips (Glass) or 15 kPa PAA gels (15 kPa). (**A, B**) Cells were exposed to either 5 mM glucose or a moderately high level of glucose (10–15 mM) for 24 h and stained with JC-1. Representative images (**A**) and quantitative results are shown (**B**). (**C, D**) Cardiomyocytes were incubated with 5 or 15 mM glucose in the absence or presence of 0.25 or 1 mM NAC for 24 h. Cells were stained with JC-1. Representative images (**C**) and quantitative results are shown (**D**). (**B, D**) For quantification, the red/green fluorescence intensity ratio of each cell was counted (n = 24 per group). Data of cells on glass coverslips treated with 5 mM glucose were set as 1.0, and data are expressed as mean ± SD. Scale bar = 50 μm. *p < 0.05, **p < 0.01. **(E)** Intracellular ATP levels after treatment of cells with 5, 15 or 25 mM glucose in the absence/presence of a ROS scavenger were measured. Intracellular ATP level in cells on glass treated with 5 mM glucose without NAC was set 100%. Data are expressed as mean ± SD (n = 5). *p < 0.05, **p < 0.01.

As ROS accumulation is known to cause mitochondrial dysfunction [23], mitochondrial membrane potential was evaluated utilizing two types of indicators; JC-1 and MitoTracker Red CMXRos. When cells were exposed to 5 mM glucose, mitochondrial membrane potential determined by JC-1 was significantly higher in cells on 15 kPa PAA gels than in cells on glass coverslips (**Fig 3A and 3B**), which is compatible with the effect of the substrate type on ROS level (**Fig 2A and 2B**). Exposure of cardiomyocytes to moderately high levels of glucose (10–15 mM) significantly decreased mitochondrial membrane potential in cells on both substrates (**Fig 3A–3D**). However, the attenuation of membrane potential caused by 15 mM glucose was markedly prevented in the presence of a ROS scavenger in cells on 15 kPa gels, whereas a scavenger failed to prevent membrane potential attenuation in cells on glass coverslips (**Fig 3C and 3D**). Similar results were obtained when MitoTracker Red CMXRos was used, instead of JC-1, to evaluate mitochondrial membrane potential **(S1A and S1B Fig)**. Furthermore, mannitol failed to decrease mitochondrial membrane potential **(S1A and S1B Fig)**, indicating that high-glucose-induced mitochondrial dysfunction is not due to altered osmolality. Prevention of high-glucose-induced mitochondrial dysfunction by a ROS scavenger was also achieved by treating cardiomyocytes on 15 kPa PAA gels with another chemically-unrelated ROS scavenger, Mn(III)tetrakis (4-benzoic acid) porphyrin (MnTBAP) **(S4 Fig)**. Mitochondrial functions were also evaluated by measuring intracellular ATP level. As shown in **Fig 3E**, cells on 15 kPa gels exhibited higher levels of ATP than cells on glass coverslips, when they were exposed to 5 mM glucose. Furthermore, cells on 15 kPa gels exhibited attenuated ATP levels when cells were treated with 15 or 25 mM glucose, which was prevented in the presence of NAC. On the other hand, a significant reduction in ATP level required 25 mM glucose for cells on glass coverslips, which was unresponsive to NAC (**Fig 3E**). Given these results, a moderately high level of glucose causes mitochondrial dysfunction, which parallels ROS accumulation and can be prevented by the presence of a ROS scavenger, in cells on mechanically compliant substrates, but not in cells on glass coverslips.

### Cardiomyocytes on glass coverslips showed enhanced apoptosis

**Fig 4:**
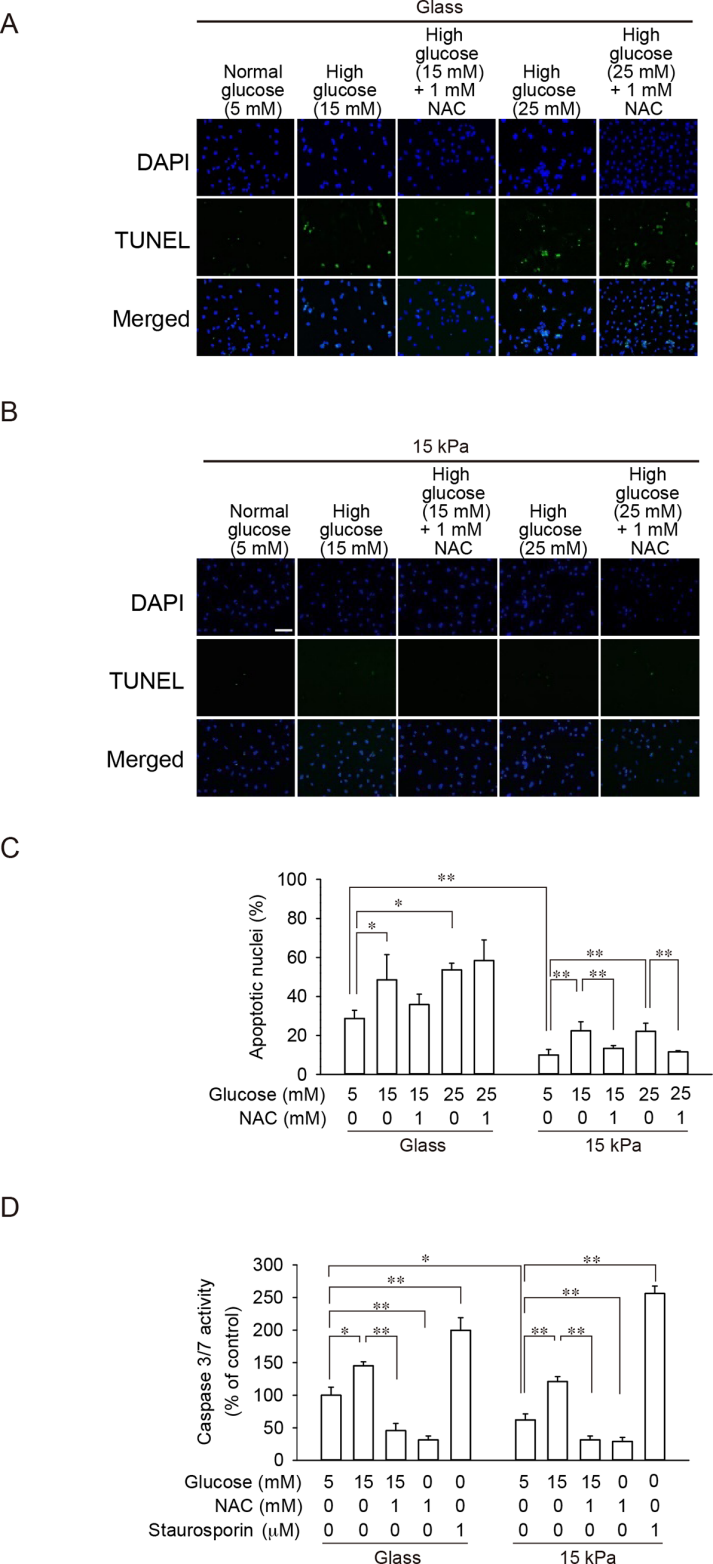
Enhanced apoptosis of cardiomyocytes cultured on glass coverslips compared with those on 15 kPa PAA gels analyzed by TUNEL assay and caspase 3/7 activity. **(A-C)** Cardiomyocytes were seeded either on glass coverslips (**A** and ‘Glass’ in **C**) or 15 kPa PAA gels (**B** and ‘15 kPa gels’ in **C**). Cells were incubated with 5, 15 or 25 mM glucose in the absence or presence of 1 mM NAC for 24 h. Cells were then subjected to TUNEL assay as described in **Materials and methods**. TUNEL staining is shown in green, and DAPI staining to visualize nuclei is shown in blue. Nuclei double-labeled with both TUNEL and DAPI were considered apoptotic nuclei (**A, B**) Representative images are shown. Scale bar = 20 μm. (**C**) The percentage of TUNEL-positive nuclei in the total number of nuclei was calculated. Data are expressed as mean ± SD (n = 4). *p < 0.05 and **p < 0.01. (**D**) Caspase-3/7 activity after treatment of cells with 5 or 15 mM glucose in the absence or presence of 1 mM NAC for 24 h was measured. As a positive control for caspase 3/7 activation, cells were treated with 1 μM staurosporin for 24 h. The activity of caspase-3/7 in cells on glass treated with 5 mM glucose without NAC was set 100%. Data are expressed as mean ± SD (n = 3). *p < 0.05, **p < 0.01.

ROS accumulation in cardiomyocytes on glass coverslips and 15 kPa PAA gels was comparable when cells were treated with 15 mM glucose, and 1 mM NAC similarly prevented ROS accumulation in cells on both substrates (**Fig 2A–2D**). However, prevention of high-glucose-induced attenuation of mitochondrial membrane potential and intracellular ATP level by a ROS scavenger was successful only in cells on 15 kPa PAA gels (**Fig 3C–3E**). As ROS level was significantly higher and mitochondrial membrane potential and intracellular ATP level were significantly lower at 5 mM glucose in cardiomyocytes on glass coverslips compared with those on 15 kPa PAA gels, we speculated that cells on glass coverslips are more stressed and are prone to cell death. To prove our hypothesis, TUNEL assays to detect DNA fragmentation due to an apoptotic process were performed. As shown in **Fig 4A–4C**, the number of apoptotic nuclei was significantly higher on glass coverslips even at 5 mM glucose. Although exposure to high glucose increased the number of apoptotic cells on both substrates, glass coverslips and 15 kPa PAA gels, the number of apoptotic nuclei on glass coverslips was still markedly higher than that on 15 kPa PAA gels. In addition, prevention of apoptosis by a ROS scavenger was observed in cells on 15 kPa PAA gels only. The effect of substrate stiffness on high-glucose-induced apoptosis was also evaluated by measuring caspase-3/7 activity. As shown in **Fig 4D**, caspase-3/7 activity was significantly higher on glass coverslips even at 5 mM glucose. Exposure to high glucose increased caspase-3/7 activity, which was prevented by a ROS scavenger on both substrates. These results suggest that cardiomyocytes on glass coverslips are more stressed even under a basal condition (5 mM glucose), and further, elevation of the glucose level may bring about an enhanced process of cell death. On the other hand, the impact of higher glucose level on cell viability on mechanically compliant substrates may be limited and reversible.

### Mitochondrial dysfunction in cardiomyocytes on 15 kPa PAA gels could be treated by scavenging ROS

**Fig 5:**
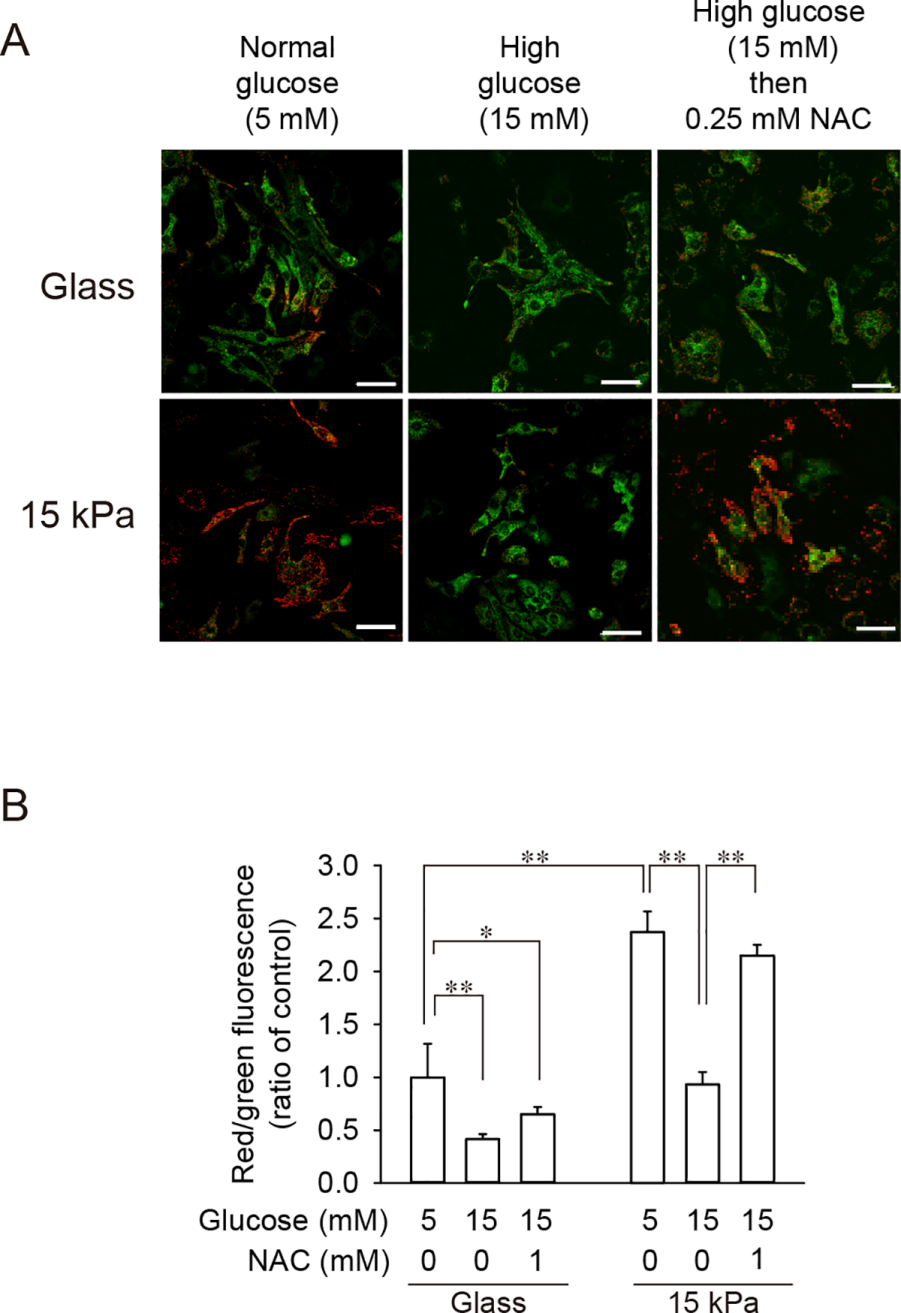
Mitochondrial dysfunction caused by moderately high glucose was restored in cells on 15 kPa PAA gels. Cardiomyocytes were seeded on either glass coverslips (Glass) or 15 kPa PAA gels (15 kPa) and exposed to 5 or 15 mM glucose for 24 h. Then cells were incubated with 5 mM glucose in the absence or presence of 0.25 mM NAC for an additional 24 h. Cells were stained with JC-1. Representative images (**A**) and quantitative results (**B**) are shown. For quantification, the red/green fluorescence intensity ratio of each cell was counted (n = 24 per group). Data of cells on glass coverslips treated with 5 mM glucose were set as 1.0, and data are expressed as mean ± SD. Scale bar = 50 μm. *p < 0.05, **p < 0.01.

Experiments to demonstrate beneficial effects of scavenging ROS on cardiomyocytes on 15 kPa PAA gels were conducted by simultaneously increasing the glucose level and adding a ROS scavenger (**Figs 2–4**). However, to establish clinical benefits of scavenging ROS, a scavenger needs to be administered subsequent to high-glucose-induced ROS accumulation. To this end, cardiomyocytes were first exposed to a high level of glucose for 24 h, followed by additional incubation with 5 mM glucose in the absence or presence of NAC. As expected, treatment of cells with 15 mM glucose significantly attenuated the mitochondrial membrane potential regardless of the substrate type (**Fig 5**). However, addition of a ROS scavenger following high-glucose treatment successfully restored the membrane potential only in cells on 15 kPa PAA gels. Given these results, scavenging ROS might have clinical significance for ameliorating cardiac dysfunction in diabetes. Furthermore, the protective effect of scavenging ROS against high-glucose-induced mitochondrial dysfunction can be successfully modeled *in vitro*, if cardiomyocytes are placed on mechanically compliant substrates.

### Regulation of glucose transport and glucose transporter expression in cells on 15 kPa PAA gels

**Fig 6:**
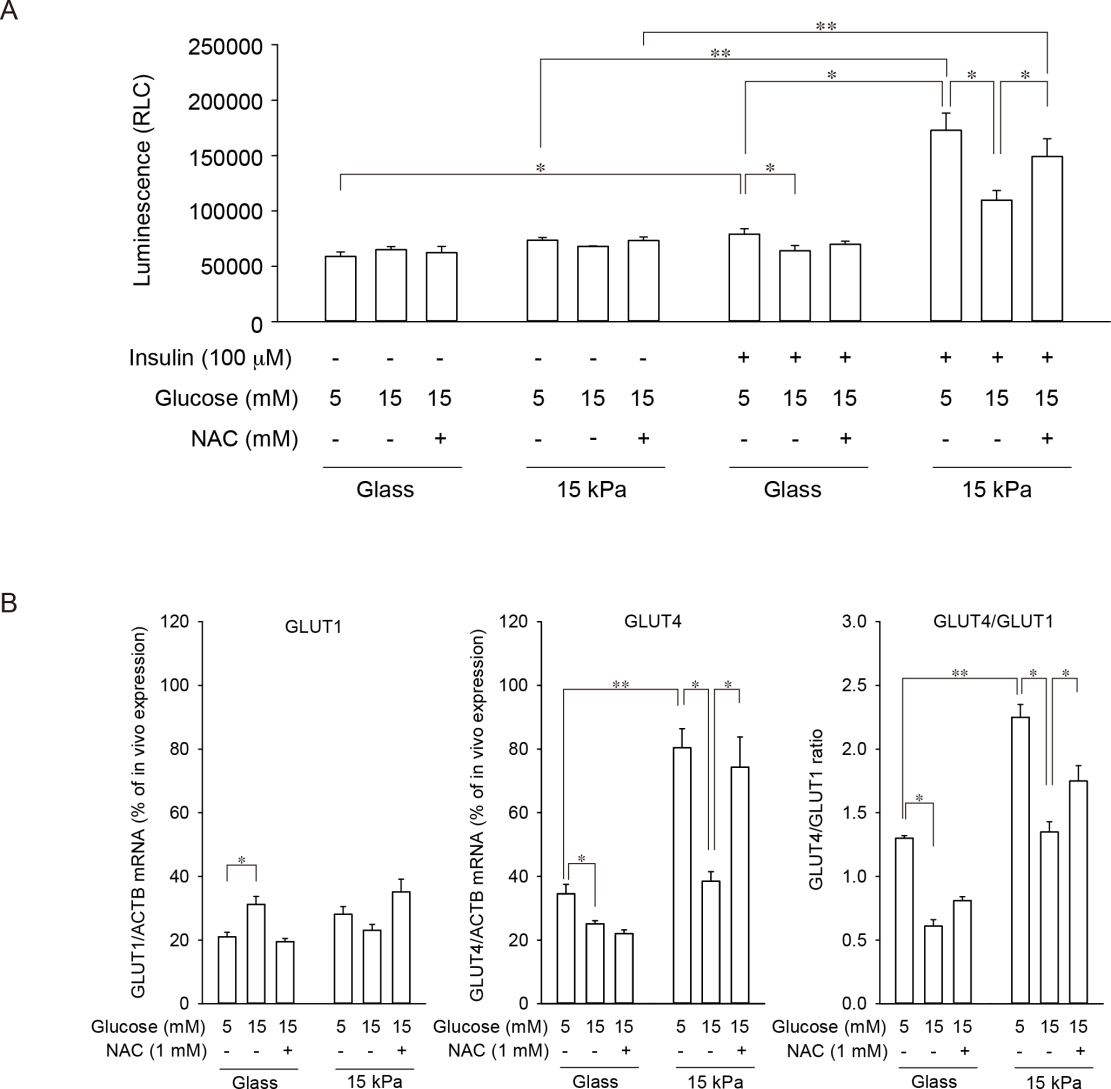
Effect of high-glucose treatment in presence or absence of ROS scavenger on insulin-stimulated glucose uptake and glucose transporter expression. Cardiomyocytes were seeded either on glass coverslips (Glass) or 15 kPa PAA gels (15 kPa). Cells were exposed to 5 or 15 mM glucose for 24 h in the absence or presence of 1 mM NAC. (**A**) Insulin-stimulated glucose uptake was measured as described in **Materials and methods**. Data are expressed as mean ± SD (n = 5). (**B**) Expression of GLUT1, GLUT4 and β-actin (ACTB) was quantified by real time PCR as described in **Materials and methods**. The ratio of GLUT1 (**Left panel**) or GLUT4 (**Middle panel**) expression to β-actin expression was evaluated, and the ratio of GLUT1/ACBT or GLUT4/ACTB observed in heart tissue isolated from three neonatal rats was set as 100%. (**Right panel**) The ratio of GLUT4 expression to GLUT1 expression is shown. Data are expressed as mean ± SD (n = 5). *p < 0.05, **p < 0.01.

Insulin-stimulated glucose uptake through GLUT4 is attenuated in diabetes, which, especially in type 2 diabetes, is one of the major pathophysiological features of cardiomyocytes [10]. Thus, the impact of high glucose in the presence or absence of a ROS scavenger on insulin-stimulated glucose uptake was compared between cardiomyocytes on glass coverslips and on 15 kPa PAA gels **(Fig 6A)**. When cardiomyocytes on glass coverslips were cultured in 5 mM glucose, insulin stimulation slightly but significantly increased glucose uptake. Although high-glucose treatment of these cells to mimic a diabetic condition significantly decreased insulin-stimulated glucose uptake, addition of NAC failed to restore attenuated glucose uptake. On the other hand, cardiomyocytes on 15 kPa gels cultured in 5 mM glucose showed markedly increased glucose uptake when stimulated with insulin. Furthermore, high-glucose treatment markedly attenuated insulin-stimulated glucose uptake and, contrary to cells on glass coverslips, NAC successfully prevented these alterations in glucose uptake caused by high-glucose treatment.

It has been reported that GLUT4 and GLUT1 are two major types of glucose transporters expressed in cardiomyocytes, and GLUT1 is responsible for glucose uptake under a basal condition, whereas GLUT4 plays a major role in insulin-stimulated glucose uptake. Their expression is influenced developmentally, as well as by the blood glucose level [24, 25]. Furthermore, as reported previously, primary cells lose their original phenotype rather quickly once they are isolated [26], which may influence glucose transporter expression, as well, on glass coverslips or 15 kPa gels. To elucidate the mechanism of altered insulin-stimulated glucose uptake in cardiomyocytes plated on glass coverslips or 15 kPa PAA gels, the expression levels of GLUT1 and GLUT4 were analyzed and compared with their expression levels *in vivo*. As reported previously [24, 25], the neonatal rat heart expresses both GLUT1 and GLUT4 **(S5 Fig)**. Both GLUT1 and GLUT4 are expressed in cardiomyocytes on glass coverslips at approximately 20–35% of the level found in the neonatal rat heart **(Fig 6B)**. High-glucose treatment slightly but significantly upregulated GLUT1, which was prevented by NAC. GLUT4 expression was downregulated by high glucose in these cells, which was unaffected by NAC. On the contrary, GLUT4 expression in cardiomyocytes cultured in 5 mM glucose on 15 kPa PAA gels was approximately 80% of the level observed in the neonatal rat heart, being much more abundantly expressed than in cells on glass coverslips. High-glucose treatment markedly downregulated GLUT4 expression, which was completely prevented by NAC. GLUT1 expression in these cells remained low (approximately 20–30% of that expressed in the neonatal rat heart), and both high-glucose treatment and NAC addition failed to show a change. These results suggest that cardiomyocytes cultured on mechanically compliant substrates express GLUT4 more abundantly than GLUT1, at a level almost comparable to that *in vivo*. Furthermore, high-glucose-induced ROS markedly downregulates GLUT4, which contributes to attenuation of insulin-stimulated glucose uptake, similarly to *in vivo* findings.

## Discussion

Cells cultured *ex vivo* have served as an easy-to-access and versatile tool to reproduce and investigate physiological and pathological events *in vivo* [27]. However, the basic assumption in cell-based assays, that cells cultured *ex vivo* exhibit similar cellular functions to their equivalent cells *in vivo*, has been challenged. Cell lines share only a part of the cellular functions of their equivalent cells *in vivo*. Primary cells lose their original phenotype rather quickly once they are isolated [26]. Neonatal rat cardiomyocytes are excellent tools for studying a wide variety of processes such as apoptosis, oxidative stress, hypertrophic growth, signal transduction, and transcriptional regulation, which could be difficult or even impossible to conduct *in vivo* [28–30]. The value of these cells has recently increased even more by co-culturing them with non-myocytes or forming three-dimensional scaffolds with various matrix proteins, which enables them to form functional cardiac tissues after several days in culture [31]. In this study, we cultured neonatal rat cardiomyocytes on substrates whose stiffness matched that of the heart. Here, we observed that high-glucose treatment disorganized the cytoskeleton **(Fig 1)**, which was prevented by scavenging ROS **(S3 Fig)**. We also observed that neonatal rat cardiomyocytes exhibited a low level of ROS, high mitochondrial membrane potential and high intracellular ATP level at a glucose level equivalent to the normal blood glucose level, only when cells were on mechanically compliant substrates (**Figs 2 and 3**).

In many studies exploring the effect of high glucose concentration, cells were exposed to 25 mM (450 mg/dl) glucose. In this study, we used 10–15 mM (180–270 mg/dL), besides 25 mM, to represent a blood glucose level that would be high enough to cause chronic complications but not high enough to cause acute metabolic disorders. Interestingly, even at 10–15 mM glucose, cells on compliant gels, but not on glass, exhibited reversible ROS accumulation and mitochondrial dysfunction without apparent apoptosis (**Figs 2–5**). Thus, application of neonatal rat cardiomyocyte culture on substrates mimicking the stiffness of their microenvironment in future studies might help elucidate a mechanistic insight into oxidative stress-induced insult on mitochondria and its contribution to the development of chronic cardiac complications in diabetes. Besides diabetic cardiac complications, cardiomyocytes on mechanically compliant substrates might also contribute to promoting research in dilated cardiomyopathy or restrictive cardiomyopathy, since histological similarities between diabetic cardiomyopathy and these forms of cardiomyopathy have been reported [32]. In fact, involvement of oxidative stress in the pathophysiology of dilated cardiomyopathy or restrictive cardiomyopathy has been suggested [33].

Multiple cellular functions depend on intact cytoskeletal structures [34–38]. Cytoskeletal structures in cardiomyocytes, such as sarcomeres and F-actin, have been reported to be regulated by both the stiffness of their substrate [14] and the glucose level [39]. In fact, these cytoskeletal structures were disorganized by high-glucose treatment in cardiomyocytes on 15 kPa gels, which was not attributable to increased osmolality **(Fig 1 and S1 Fig)**. Nevertheless, alteration of cytoskeletal structures by high-glucose treatment was not observed in cells on glass coverslips. The exact cause of the cytoskeletal structure on glass not responding to high glucose is not clear at this time. However, previous reports suggest that mechanical input from the substrate regulates cytoskeletal structures similarly to biochemical signals in other cell types [19]. Thus, it could be possible that a mechanical signal from unphysiologically stiff glass overrode a biochemical signal to disorganize cytoskeletal structures originated from high glucose. It is of interest that high-glucose treatment induced structural alteration of sarcomeres, as this may lead to contractile dysfunction. In fact, ROS-induced contractility dysfunction was reported in a type 1 diabetes model [40], and structural alteration of the cardiac Z-disc caused by high glucose has been implicated in the development of cardiac dysfunction [41]. Further research is necessary to elucidate the association between disorganized cytoskeletal structures and contractility dysfunction in high glucose, using cardiomyocytes on mechanically compliant substrates.

Besides substrate stiffness, cardiomyocytes are subjected to other mechanical input from their microenvironment *in vivo*, as well, since hearts are dynamically contracting and relaxing to maintain blood circulation. Such dynamic mechanical loading has been shown to influence cardiomyocyte functions [42, 43]. Although cardiomyocytes on 15 kPa gels exhibited spontaneous beating (data not shown), which strained the polyacrylamide gels underneath the cells, a previous study has reported that the stiffness of polyacrylamide, unlike that of native tissues, would be unchanged by strain caused by spontaneous beating [44]. Thus, the mechanical input from 15 kPa polyacrylamide gels to cardiomyocytes is rather static, as opposed to dynamic, which cardiomyocytes would face *in vivo*. However, even without dynamic mechanical alterations, cardiomyocytes on 15 kPa gels exhibited higher susceptibility to high glucose under ROS-induced mitochondrial dysfunction and attenuated insulin-stimulated glucose uptake, which diabetic cardiomyocytes would face *in vivo*. Therefore, culturing cardiomyocytes on 15 kPa polyacrylamide gels would be sufficient to reproduce some key events observed in diabetic cardiomyocytes. Further research including the impact of dynamic mechanical loading on cardiomyocytes and elucidating its underlying mechanism would contribute to improving the accuracy of modeling *in vivo* cardiomyocytes *ex vivo*.

Glucose transporter expression in cardiomyocytes is influenced by development, and the predominant type switches from GLUT1 to GLUT4 [24, 25]. Since neonatal rat cardiomyocytes express abundant GLUT1 compared with human cardiomyocytes, adopting these cells as a model of human cardiomyocytes has been challenged. In fact, expression levels of GLUT1 and GLUT4 were similar in neonatal rat heart tissues (**S5 Fig**). However, cardiomyocytes cultured on 15 kPa gels, but not cells on glass coverslips, expressed significantly more GLUT4 than GLUT1 (**Fig 6B**). Thus, neonatal cardiomyocyte culture on mechanically compliant substrates could make these cells more suitable for modeling insulin resistance under a high glucose environment than neonatal cardiomyocyte culture on conventional but unphysiologically stiff substrates.

In conclusion, by placing cells on mechanically compliant substrates, we have developed an *in vitro* cardiomyocyte culture, which could reproduce *in vivo* cellular events caused by high glucose at a range capable of causing chronic diabetic complications in the heart. This study is significant as these cells could serve as an innovative tool for a vast and unexploited area of research on the diabetic heart *in vitro*.

## Supporting information

S1 Fig Disorganized cytoskeleton and attenuated mitochondrial membrane potential caused by high-glucose treatment were independent of alteration in osmolality. Cardiomyocytes were seeded on either glass coverslips (Glass) or 15 kPa PAA gels (15 kPa) and treated with either 5 mM glucose, 15 mM glucose or 15 mM mannitol for 24 h. **(A)** Representative images of cardiomyocytes stained for α-actinin (green), mitochondrial membrane potential with MitoTracker Red CMXRos (red), and DAPI (blue). Scale bar = 20 μm **(B)** Fluorescence intensity of Mito Tracker Red CMXRos shown in **(A)** was quantified (n = 24 per group). Fluorescence intensity of cells on glass coverslips treated with 5 mM glucose was set as 100%, and data are expressed as mean ± SD (n = 24). *p < 0.05, **p < 0.01.
(TIF)

S2 Fig ROS accumulation induced by high-glucose treatment was independent of alteration in osmolality. Cardiomyocytes were seeded on either glass coverslips (Glass) or 15 kPa PAA gels (15 kPa) and treated with either 5 mM glucose, 10 mM glucose, 15 mM glucose 10 mM mannitol or 15 mM mannitol for 24 h and stained with H_2_DCFDA. Representative images **(A)** and quantitative results are shown **(B)**. Fluorescence intensity in cells on glass coverslips treated with 5 mM glucose was set as 100%, and data are expressed as mean ± SD (n = 24). Scale bar = 50 μm. *p < 0.05, **p < 0.01 vs. control (5 mM glucose group on glass).
(TIF)

S3 Fig Cytoskeletal disorganization due to high-glucose treatment was prevented by scavenging ROS in cardiomyocytes on 15 kPa gels, but not in cardiomyocytes on glass coverslips. Cardiomyocytes were seeded on either glass coverslips (Glass) or 15 kPa PAA gels (15 kPa) and treated with 5 or 15 mM glucose in the absence or presence of 1 mM NAC for 24 h. Sarcomere structures were identified by staining for α-actinin (red), F-actin structures were stained with phalloidin (green), and nuclei were stained with DAPI (blue). Representative images are shown. Scale bar = 20 μm.
(TIF)

S4 Fig Attenuated mitochondrial membrane potential due to high-glucose treatment was prevented by a scavenger, MnTBAP, in cardiomyocytes on 15 kPa gels, but not in cardiomyocytes on glass coverslips. Cardiomyocytes were seeded on either glass coverslips (Glass) or 15 kPa PAA gels (15 kPa) and treated with 5 or 15 mM glucose in the absence or presence of 0.1 mM MnTBAP for 24 h. Cells were stained with JC-1. Representative images are shown. Scale bar = 50 μm.
(TIF)

S5 Fig Expression of GLUT1 or GLUT4 in neonatal rat heart. Three neonatal rat hearts were homogenized and combined. Expression of GLUT1, GLUT4 and β-actin (ACTB) was quantified by real time PCR as described in **Materials and methods**. The ratio of GLUT1 or GLUT4 expression to ACTB expression is shown.
(TIF)

## Abbreviations

PAA: polyacrylamide
ROS: reactive oxygen species
GLUT: glucose transporter
H_2_DCF-DA: 2',7'-dichlorodihydrofluorescein diacetate
JC-1: 5,5',6,6'-tetrachloro-1,1',3,3'-tetraethylbenzimidazolocarbocyanine iodide
DAPI: 4',6-diamidino-2-phenylindole dihydrochloride
ECM: extracellular matrix
NAC: N-acetyl cysteine
