# Supplementary figures and images for: Cardiomyocytes cultured on mechanically compliant substrates, but not on conventional culture devices, exhibit prominent mitochondrial dysfunction due to reactive oxygen species and insulin resistance under high glucose
Source: PLoS One. 2018 Aug 23;13(8):e0201891. doi: 10.1371/journal.pone.0201891 (PMC6107143; doi:10.1371/journal.pone.0201891)

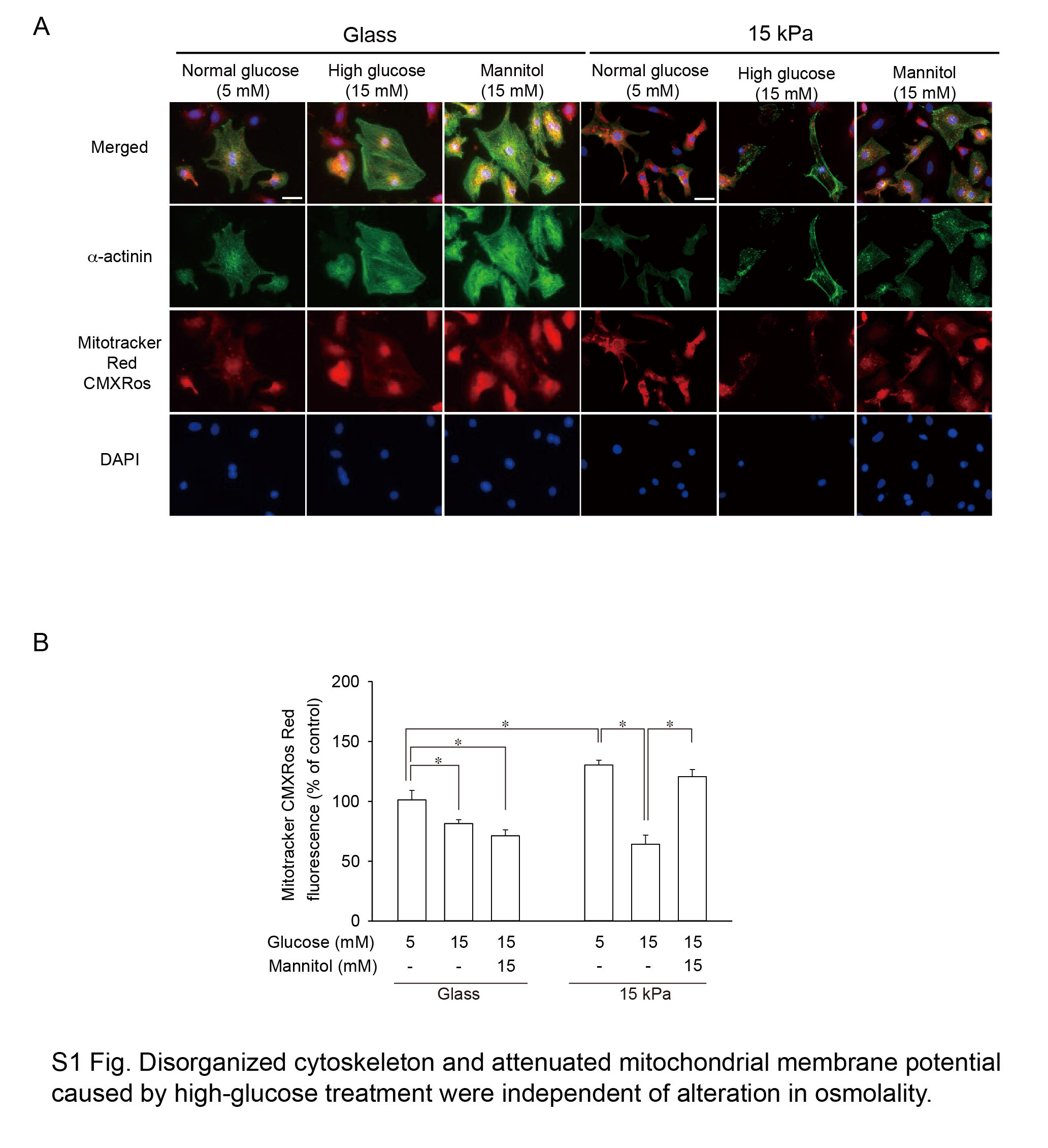

Supplement: S1 Fig — Cardiomyocytes were seeded on either glass coverslips (Glass) or 15 kPa PAA gels (15 kPa) and treated with either 5 mM glucose, 15 mM glucose or 15 mM mannitol for 24 h. (A) Representative images of cardiomyocytes stained for α-actinin (green), mitochondrial membrane potential with MitoTracker Red CMXRos (red), and DAPI (blue). Scale bar = 20 μm (B) Fluorescence intensity of Mito Tracker Red CMXRos shown in (A) was quantified (n = 24 per group). Fluorescence intensity of cells on glass coverslips treated with 5 mM glucose was set as 100%, and data are expressed as mean ± SD (n = 24). *p < 0.05, **p < 0.01. (TIF) [file pone.0201891.s001.tif]

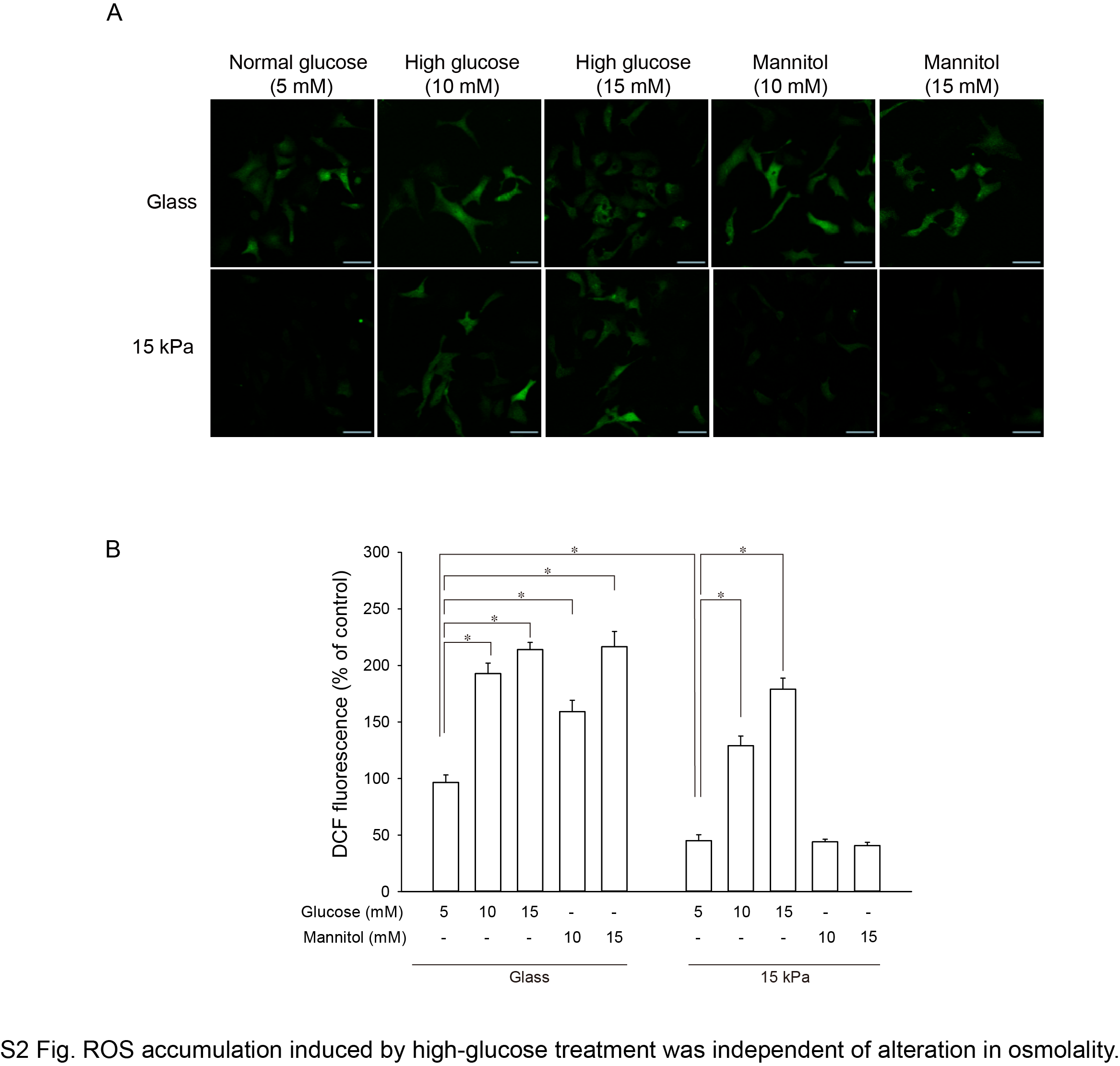

Supplement: S2 Fig — Cardiomyocytes were seeded on either glass coverslips (Glass) or 15 kPa PAA gels (15 kPa) and treated with either 5 mM glucose, 10 mM glucose, 15 mM glucose 10 mM mannitol or 15 mM mannitol for 24 h and stained with H2DCFDA. Representative images (A) and quantitative results are shown (B). Fluorescence intensity in cells on glass coverslips treated with 5 mM glucose was set as 100%, and data are expressed as mean ± SD (n = 24). Scale bar = 50 μm. *p < 0.05, **p < 0.01 vs. control (5 mM glucose group on glass). (TIF) [file pone.0201891.s002.tif]

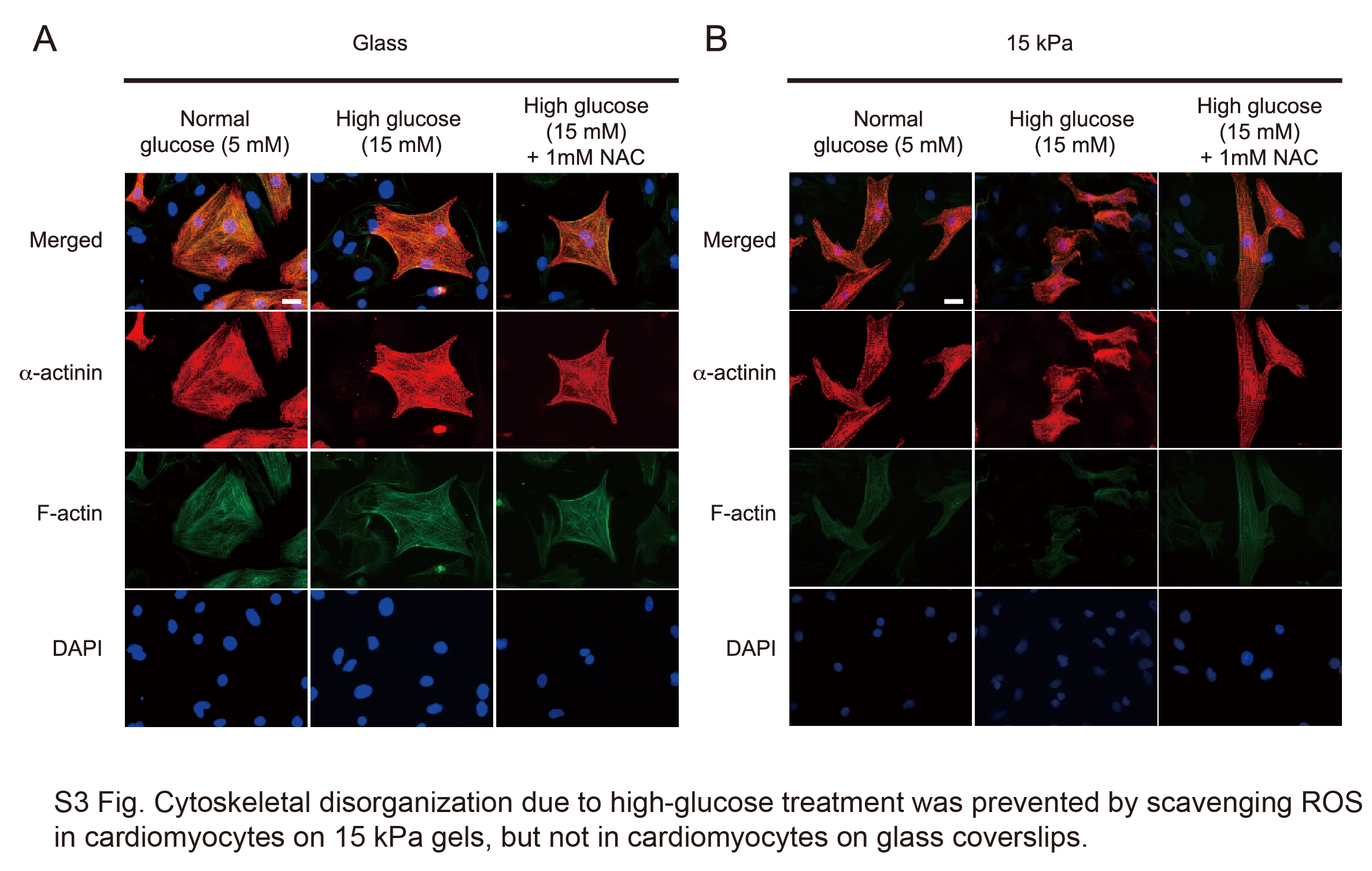

Supplement: S3 Fig — Cardiomyocytes were seeded on either glass coverslips (Glass) or 15 kPa PAA gels (15 kPa) and treated with 5 or 15 mM glucose in the absence or presence of 1 mM NAC for 24 h. Sarcomere structures were identified by staining for α-actinin (red), F-actin structures were stained with phalloidin (green), and nuclei were stained with DAPI (blue). Representative images are shown. Scale bar = 20 μm. (TIF) [file pone.0201891.s003.tif]

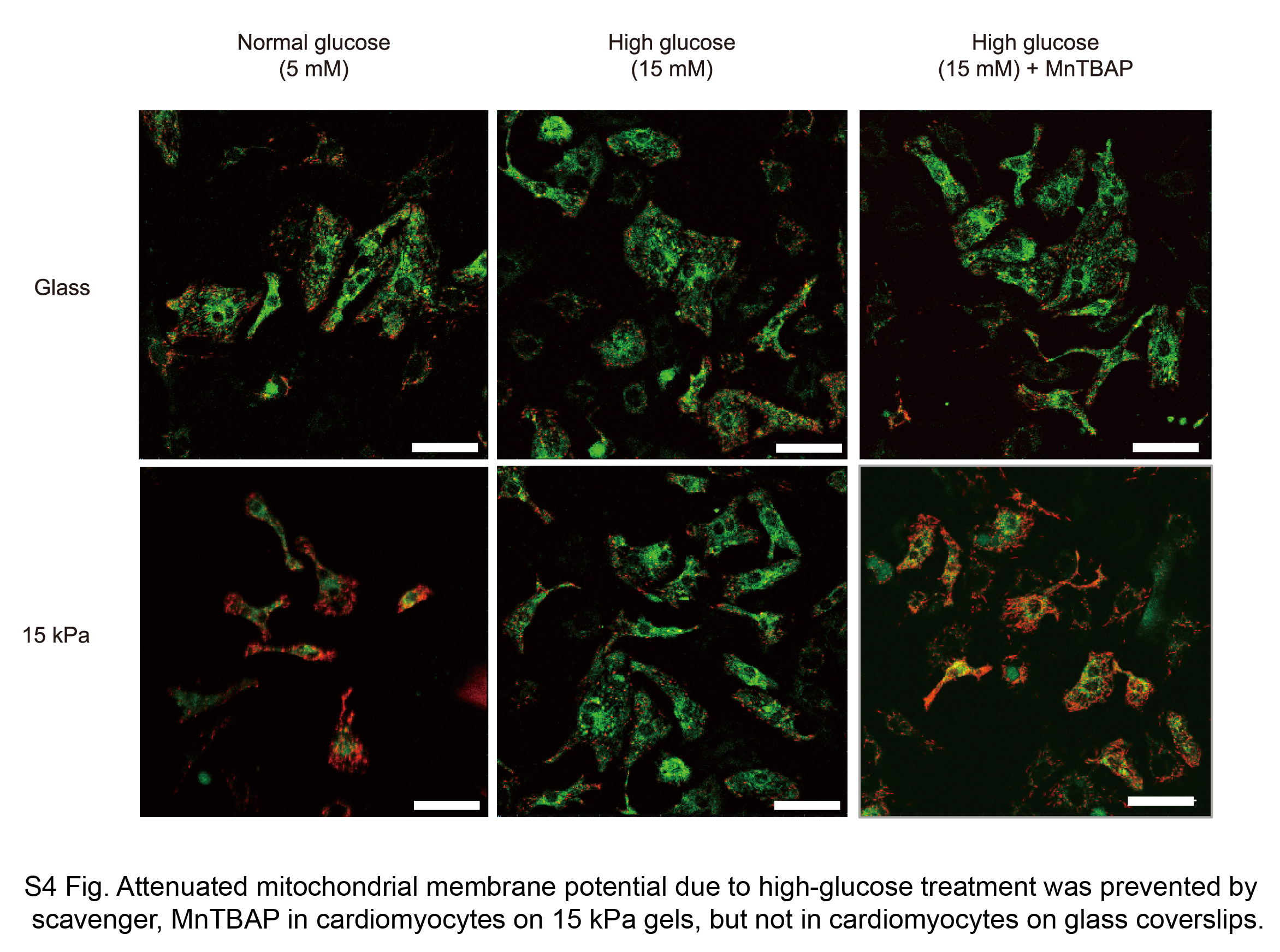

Supplement: S4 Fig — Cardiomyocytes were seeded on either glass coverslips (Glass) or 15 kPa PAA gels (15 kPa) and treated with 5 or 15 mM glucose in the absence or presence of 0.1 mM MnTBAP for 24 h. Cells were stained with JC-1. Representative images are shown. Scale bar = 50 μm. (TIF) [file pone.0201891.s004.tif]

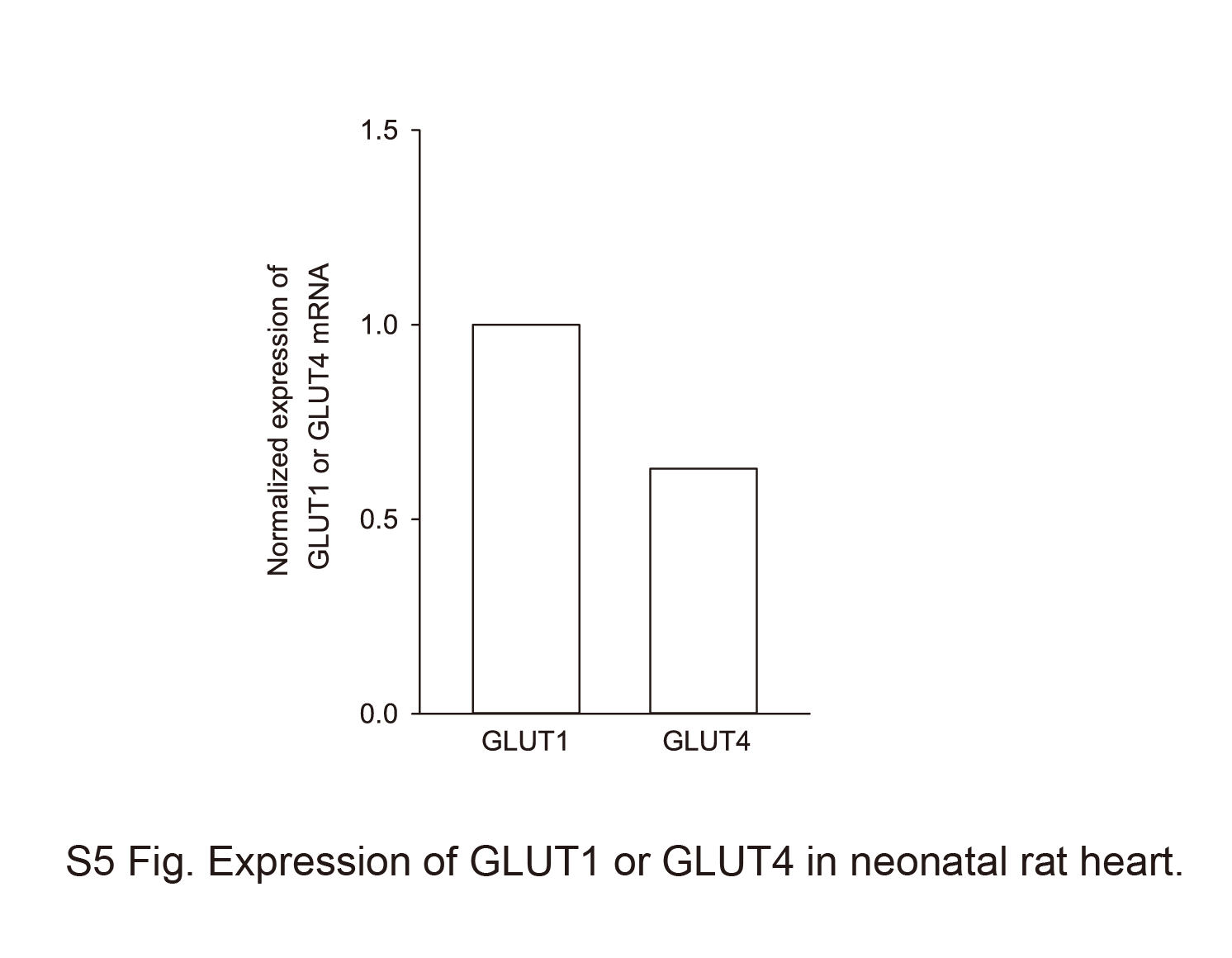

Supplement: S5 Fig — Three neonatal rat hearts were homogenized and combined. Expression of GLUT1, GLUT4 and β-actin (ACTB) was quantified by real time PCR as described in Materials and methods. The ratio of GLUT1 or GLUT4 expression to ACTB expression is shown. (TIF) [file pone.0201891.s005.tif]
